# Substoichiometric inhibition of transthyretin misfolding by immune-targeting sparsely populated misfolding intermediates: a potential diagnostic and therapeutic for TTR amyloidoses

**DOI:** 10.1038/srep25080

**Published:** 2016-04-28

**Authors:** Natalie J. Galant, Antoinette Bugyei-Twum, Rishi Rakhit, Patrick Walsh, Simon Sharpe, Pharhad Eli Arslan, Per Westermark, Jeffrey N. Higaki, Ronald Torres, José Tapia, Avijit Chakrabartty

**Affiliations:** 1 Princess Margaret Cancer Centre, University Health Network, Departments of Medical Biophysics and Biochemistry, University of Toronto, TMDT 4-305, 101 College Street, Toronto, Ontario, Canada M5G 1L7; 2 Department of Chemical and Systems Biology, Stanford University, CA 94305, USA; 3 Molecular Structure and Function Program, the Hospital for Sick Children, Department of Biochemistry, University of Toronto, 1 King’s College Circle, Toronto, Ontario, Canada M5S 1A8; 4 Department of Immunology, Genetics and Pathology, Uppsala University, Uppsala, Sweden; 5 Departments of Biochemistry and Histopathology, Prothena Biosciences Inc., South San Francisco, CA 94080.

## Abstract

Wild-type and mutant transthyretin (TTR) can misfold and deposit in the heart, peripheral nerves, and other sites causing amyloid disease. Pharmacological chaperones, Tafamidis^®^ and diflunisal, inhibit TTR misfolding by stabilizing native tetrameric TTR; however, their minimal effective concentration is in the micromolar range. By immune-targeting sparsely populated TTR misfolding intermediates (i.e. monomers), we achieved fibril inhibition at substoichiometric concentrations. We developed an antibody (misTTR) that targets TTR residues 89–97, an epitope buried in the tetramer but exposed in the monomer. Nanomolar misTTR inhibits fibrillogenesis of misfolded TTR under micromolar concentrations. Pan-specific TTR antibodies do not possess such fibril inhibiting properties. We show that selective targeting of misfolding intermediates is an alternative to native state stabilization and requires substoichiometric concentrations. MisTTR or its derivative may have both diagnostic and therapeutic potential.

Protein misfolding diseases constitute a significant health care burden, especially in terms of their economic impact and associated costs of care^1^,^2^. Development of effective therapies for protein misfolding diseases requires an understanding of how proteins change from their natural conformation to misfolded pathological forms. In their natural state, proteins can be either structured or intrinsically disordered to perform their physiological function^3^. For those proteins that adopt a unique three-dimensional structure, which in most cases corresponds to the lowest free energy conformation, mutations or changes in environmental conditions can result in destabilization of this low energy state, and lead to misfolding, aggregation, and/or degradation^4^; this misfolding ultimately results in the development of protein misfolding disease.

Although a number of approved drugs for protein misfolding disease are available, they mainly offer symptomatic relief with few addressing the underlying root cause^5^. Protein misfolding diseases are caused by a multi-step pathway that results in the conversion of native proteins into abnormal conformations that lead to fibril formation and aggregation. The misfolding pathway can in many cases be described by a nucleation and growth model^6^ or rather a downhill polymerization reaction model^7^. The native protein must first overcome an energetic barrier to populate transient high-energy intermediates. Conversion of these intermediates to aggregates/fibrils is an energetically favorable process, and pre-formed aggregates can ‘seed’ the conversion of other native proteins into the misfolded form. There are several conceptual strategies available for intervention that target different stages in the protein misfolding pathway (Fig. 1A).

Although the native and fibrillar states are the most stable and more populated species under steady-state conditions, the sparsely populated oligomeric intermediates are the ones which have been directly implicated in cytotoxicity (*vida infra*)^8^. It follows that effective treatments should reduce the toxic oligomeric intermediates. Current strategies in development include native state stabilization, fibril capping, and aggregate sequestration. However, these only indirectly affect the putative toxic oligomeric intermediates, and mostly target species in the misfolding pathway present at high concentrations, namely the native and fibrillar states.

Native state stabilization strategies utilize small molecules called pharmacological chaperones that prevent aggregation by binding to the native protein, which imposes an energetic barrier to the formation of aggregation-prone misfolding intermediates. Pharmacological chaperones must possess sufficient binding free energy such that they alter the folding equilibrium to de-populate aggregation-prone species on the misfolding pathway. An alternative approach to native-state stabilization that limits aggregation involves capping the ends of growing amyloid fibrils with specific peptides or small molecules^9^,^10^. However, capping aggregates results in the build-up of oligomers and small aggregates which can worsen the problem since oligomeric intermediates have often been shown to be more toxic than the final fibrillar end-product^11^. The concentration of toxic oligomeric species can be reduced by accelerating the aggregation process, as shown when small molecules were used to convert toxic Alzheimer amyloid peptide oligomers into less toxic amyloid fibrils^12^. Small-molecule pharmacological chaperones that stabilize the native state remain the strategy with a relatively good track record of development.

Native tetramer stabilization has been the main approach used to design therapy for a group of protein misfolding diseases caused by the same amyloid-precursor protein, transthyretin. Misfolding of transthyretin (TTR), a human serum transport protein, is the root cause of a group of amyloidoses for which no FDA approved treatment currently exists. TTR is an abundant homotetrameric protein which transports thyroid hormones, T3 and T4, and Vitamin A (in a ternary complex with retinol binding protein) throughout the body^13^. Its primary site of production is the liver, which secretes it into the serum. Secondary sites include the retinal pigment epithelia and the choroid plexus which secretes it into the vitreous humour and cerebrospinal fluid, respectively^14^. Aberrant dissociation of the TTR tetramer into its misfolded monomers leads to formation and deposition of amyloid fibrils at multiple sites throughout the body; these amyloid deposits contain both full-length TTR and proteolytic fragments of TTR^15^. It is believed that the dissociation pathway of the native TTR tetramer is a multi-step process in which the TTR protein cycles through a series of misfolding intermediates before becoming an insoluble amyloid^16^. Although the mechanism of TTR misfolding is uncertain, studies with transgenic mice which over-express TTR suggest that it may be the result of complications either in TTR’s initial folding stages due to defective hepatic chaperones, or during its final stages of clearance as a result of defective proteasomes^17^.

There are two main conditions related to the misfolding of the TTR protein: wild-type TTR amyloidosis (wt-ATTR) and hereditary forms of TTR amyloidosis^18^. wt-ATTR is characterized by wild-type TTR amyloid deposition, while the latter is a heterogeneous group of autosomal dominant diseases caused by mutation in the TTR gene. wt-ATTR involves amyloid deposition in the heart that can result in cardiac insufficiency and death, with secondary deposition in the peripheral nervous system and eye^19^. Deposition of wild-type TTR amyloid can also occur in both cartilage and ligaments and is believed to be the cause of other debilitating diseases such as lumbar spinal stenosis^20^. Additional wt-ATTR co-morbidities include bilateral carpal tunnel syndrome, which is believed to be one of the first signs of wt-ATTR as it precedes wt-ATTR diagnosis by 3–5 years^21^,^22^.

There have been more than 125 different TTR mutations identified in various populations worldwide^23^. The phenotypic expression of these mutations varies but most often results in the development of systemic amyloidosis. A slowly progressing axonal sensory autonomic and motor neuropathy, often called ‘familial amyloidotic polyneuropathy (FAP) or restrictive cardiomyopathy are major consequences^24^. In many cases there is a mixture of these cardinal symptoms.

Current therapeutic strategies used in TTR amyloidosis involve surgical procedures that either remove amyloid infiltrated tissue (i.e. heart transplants) or decrease the concentration of circulating amyloid-prone TTR protein (i.e. liver transplants)^22^. Two recently developed small molecule agents which have successfully displayed *in vitro* inhibition of fibril formation are diflunisal and Tafamadis^®^. Both drugs are similar in their strategy of tetrameric dissociation inhibition by acting as pharmacological chaperones which directly bind to TTR’s thyroxine binding site at its dimer-dimer interface.

Although diflunisal has been demonstrated to effectively stabilize TTR structure in patients with TTR cardiac amyloidosis, its administration unfortunately compromises glomerular filtration rates and platelet counts^22^. Because diflunisal is an NSAID, administration to elderly patients, especially those with TTR cardiac complications, is contraindicated as a result of its side-effects such as renal failure, peptic ulcer disease, gastritis, and fluid retention^22^. Tafamadis^®^, a benzoxazole derivative, binds to the thyroxine binding pocket, with two molecules of Tafamadis^®^ per tetramer. While Tafamadis has no NSAID-associated side effects, near-stoichiometric concentrations (serum TTR ~3.6 μM) would be required in order to effectively reach binding saturation and prevent misfolding^25^. Drug binding leads to tetramer stabilization due to changes in the unfolding equilibrium, and the degree of this stabilization is proportionate to the drug binding free energy. The increase in native state stability leads to reduced concentrations of the unfolded state, lower protein degradation rates, and increased half-life of the native protein^26^. Because Tafimidis^®^ binds TTR relatively weakly, with an apparent dissociation constant in the micromolar range^27^, the degree of stabilization is not likely to be large. While Tafamidis^®^ has been shown to be a clinically effective pharmacological chaperone in slowing disease progression, this strategy is limited by the need for high drug concentrations, the need for saturation binding to prevent misfolding, tighter binding to confer greater stabilization, and competition with endogenous ligands that may interfere with the protein’s normal function. Although the U.S. Food and Drug Administration (FDA) has indicated that the New Drug Application (NDA) for Tafamidis^®^ has not yet been approved^28^, it has received approval for treatment of TTR amyloidosis in Europe, and has been used to treat TTR-FAP in Mexico, Japan, and Argentina^29^.

While small molecule drugs like Tafamidis^®^ have the advantages of relative ease of synthesis and high oral bioavailability, macromolecular antibody drugs have other advantages. These include their more specific mechanisms of action than conventional chemotherapeutic agents which often result in fewer associated side effects. They trigger complement-mediated processes that scavenge biological materials. Furthermore, humanized monoclonal antibodies, which are the most common protein drugs entering clinical studies, have higher drug approval rates^30^ than new small-molecule drugs^31^.

We used a structure-based approach to develop a TTR conformation-specific antibody that targets the pre-fibrillar, misfolding intermediates of TTR, but not the natively folded protein. This antibody directly targets the sparsely populated non-native misfolding intermediates and can achieve substoichiometric inhibition of fibril formation at nanomolar concentrations.

## Results and Discussion

### Development and validation of misTTR antibody

The use of antibodies, particularly as structural probes, to investigate the misfolding pathway of amyloidogenic and non-amyloidogenic proteins has been applied in the study of ALS^32^, prion disease^33^ and Alzheimer’s disease^34^. The generation of TTR antibodies have often relied on highly destabilized mutant proteins or randomly generated linear sequences of the molecule^15^,^35^,^36^. Using a high resolution X-ray crystal structure of TTR, we sought to develop a conformation-specific antibody which could not only be used as a structural probe of TTR misfolding intermediates, but also as a diagnostic tool for the identification of TTR-related amyloidosis patients, and perhaps even as a therapeutic agent.

**Figure 1:**
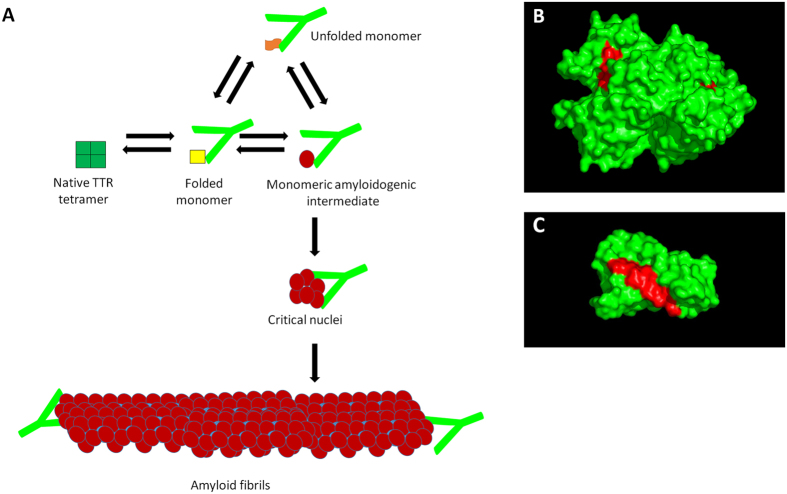
(**A**) Proposed model for the inhibition of TTR fibrillogenesis by the misTTR antibody. During the fibril formation process, native tetrameric TTR first dissociates into an altered monomeric intermediate. This altered monomeric intermediate self assembles into stable nuclei, which allows for fibril formation to proceed. Substoichiometric amounts of the misTTR antibody may suppress TTR fibril formation by binding misfolded conformations of the TTR that comprise the critical nuclei, which are likely present at very low concentrations. The misTTR antibody may also act to inhibit TTR fibrillogenesis by binding to the unfolded monomers and/or to the extremities of fibrils, essentially capping the ends of preformed fibrils to prevent fibril growth. (**B**) Surface representation of native tetrameric TTR (green) with buried misTTR epitope in red. (**C**) Surface representation of monomeric TTR (green) with exposed epitope in red. Structures were generated using PDB ID 1DVQ in Deep View (Swiss-PBD Viewer 3.7).

Examination of the X-ray crystal structure of TTR (PDB code: 3KGU) revealed that residues 89–97 was the longest segment of TTR that was highly buried (>90%) in the native tetrameric fold of the protein (Fig. 1B). These residues are located in the F-strand that is sequestered at the dimer interface of TTR and inaccessible in the native tetramer. We reasoned that an antibody targeted against this epitope would bind to misfolded TTR conformations with disrupted and exposed dimer interfaces, but not to the native tetramer. This antibody would bind to both monomeric (Fig. 1C) and non-native oligomeric forms of TTR. Since these misfolded species represent only a fraction of serum TTR, this may result in substoichiometric concentrations of the antibody being sufficient for activity. In our strategy, antibody specificity for the misfolded conformation is designed from the outset rather than screened from large libraries.

To generate such an antibody, we first synthesized a multiple antigenic peptide (MAP) where each branch of the dendritic core contained the sequence ggEHAEVVFTAggkg; the capitalized sequences represent residues 89–97 of TTR. Gly-Lys linkers were added to the N and C termini so the peptide epitope would resemble an internal sequence as well as to increase the molecular weight of the MAP antigen for an enhanced immune response. Rabbit anti-sera produced from immunization with the MAP antigen were affinity purified and used in all subsequent experiments described herein. We named this antibody, which was designed to specifically target the misfolded conformations of the TTR protein, ‘misTTR’.

**Figure 2:**
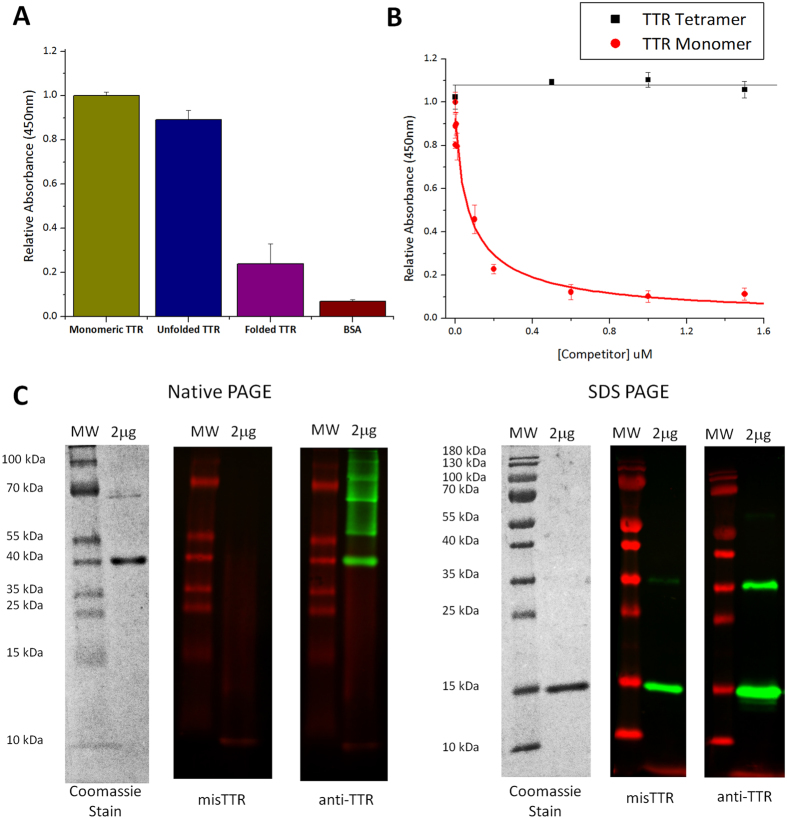
MisTTR antibody selectively binds monomeric, misfolded conformations of TTR. (**A**) Indirect ELISA using monomeric TTR (0.01 mg/mL TTR incubated at 25 °C , pH 3, 44 h), guanidine unfolded TTR (TTR in 6 M guanidine , incubated overnight at 25 °C), folded native TTR and BSA. (**B**) Competition ELISA using guanidine unfolded TTR as bound antigen, with monomeric and tetrameric TTR as competitors, and misTTR as the antibody. IC_50_ = 63 ± 20 nM for monomeric TTR’s binding affinity for misTTR. (**C**) Native and SDS-PAGE analysis was performed on wild-type TTR purified from human plasma (purchased from Sigma-Aldrich). For native PAGE analysis, three pairs of sample lanes were run. The first lane consisted of molecular weight markers while the second lane consisted of 2 mg of wild-type TTR. The three pairs of lanes were excised from the gel. The first excised lane pair was stained with Coomassie Blue dye. The second pair was transferred to PDVF membrane and immunoblotted with misTTR antibody. The third pair was similarly transferred to PDVF membrane but immunoblotted with commercial anti-TTR antibody. The same procedure was repeated for an SDS-PAGE counterpart. In the native gels, misTTR did not recognize any species, while anti-TTR recognized putative dimers and higher molecular weight oligomers. In SDS gels misTTR only recognized monomers, while anti-TTR recognized monomers and putative dimers.

To examine the binding specificity of the misTTR antibody, we tested its reactivity with folded tetrameric TTR and monomeric guanidine hydrochloride (GdnHCl) unfolded TTR^37^ using indirect and competitive enzyme-linked immunosorbent assay (ELISA). Our antibody reacts preferentially with monomeric or unfolded TTR compared to native folded TTR in an indirect ELISA (Fig. 2A). We tested the specificity of misTTR in a competition ELISA, where native and monomeric TTR in solution was tested for their ability to compete for binding to unfolded TTR absorbed to the ELISA plate. Native tetrameric TTR in solution was unable to compete (Fig. 2B). Using previously established conditions to generate the monomeric amyloidogenic intermediate^37^,^38^ we found that the misTTR antibody was able to bind to this intermediate with an IC_50_ value of 63 ± 20 nM (Fig. 2B), suggesting that substoichiometric concentrations are sufficient for effective binding. We note that this is not precisely a binding constant because while the misTTR antibody appears to be mono-specific, it is purified from a polyclonal mixture. As a control, the assay was also performed using a commercially available pan-specific anti-TTR antibody (Sigma-Aldrich) that was raised against natively folded TTR protein. As expected, natively folded TTR was able to compete for antibody binding when this antibody was used (Supplementary Figure S1). The lack of binding by the misTTR antibody to native TTR supports the concept that the selected epitope is indeed buried within the native fold of the protein as predicted from the crystal structure. This also supports our rational design strategy of targeting an epitope accessible for binding only in the misfolded TTR species, and avoiding interactions with normal, natively folded TTR that is also present at high concentrations in patient serum.

Our indirect and competition ELISA results comparing the reactivity of misTTR with folded and monomer/unfolded TTR suggest that our antibody has significant conformational specificity for misfolded TTR. To determine the oligomerization states of TTR bound by misTTR antibody we performed native and denaturing Western blots (Fig. 2C). The pan-specific commercial TTR antibody recognizes native tetrameric TTR, monomeric TTR, and an SDS-resistant TTR dimer in the denaturing Western blot (~35 kDa), whereas the misTTR antibody recognizes only monomeric (~15 kDa) TTR without recognizing native tetrameric TTR (Fig. 2C). The misTTR antibody thus fulfills our design criteria of developing an antibody with very high conformational specificity for misfolded TTR and with reasonable binding strength.

### MisTTR antibody recognizes TTR amyloid fibrils formed *in vitro* and in human tissue

**Figure 3:**
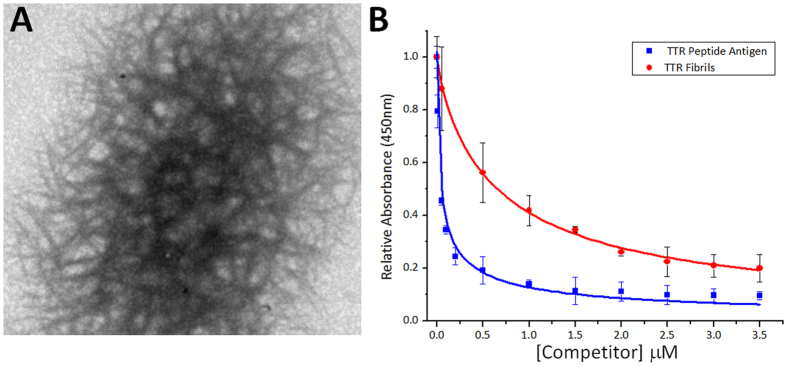
Recognition of TTR amyloid fibrils by misTTR antibody. (**A**) Representative TEM image of amyloid fibrils produced from 1 mg/mL TTR. (**B**) Competition ELISA with misTTR antibody showing selective binding of TTR amyloid fibrils and antigenic peptide.

We went on to evaluate the reactivity of the misTTR antibody with TTR amyloid fibrils formed *in vitro*. To do this, we selected physiologically relevant conditions under which TTR readily misfolds and forms amyloid fibrils (pH 4.5, 37 °C, 3 days)^39^. The aggregates formed under these conditions were able to bind the amyloid specific dye thioflavin T (ThT), causing an enhancement in the fluorescence emission of the dye as well as a characteristic red shift in its emission spectrum (Supplementary Figure S2). The presence of TTR amyloid fibrils under these conditions was also confirmed using negative stain transmission electron microscopy (TEM) (Fig. 3A). The fibrils observed were found to be ~7 nm in diameter and the morphology consistent with previously published reports of fibrillar structures formed by other amyloidogenic proteins^8^,^40^. To determine whether the misTTR antibody would recognize these TTR fibrils formed *in vitro*, a competition ELISA was performed. TTR fibrils were able to compete with plate-bound GdnHCl-unfolded TTR for binding to misTTR (Fig. 3B). Thus the misTTR antibody was able to selectively bind not only to monomeric, but to fibrillar TTR as well. Interestingly, addition of antibody during the early stages of fibrillogenesis resulted in reduction of total TTR aggregation, but did not affect the morphology of fibers formed (Supplementary Figure S3).

**Figure 4:**
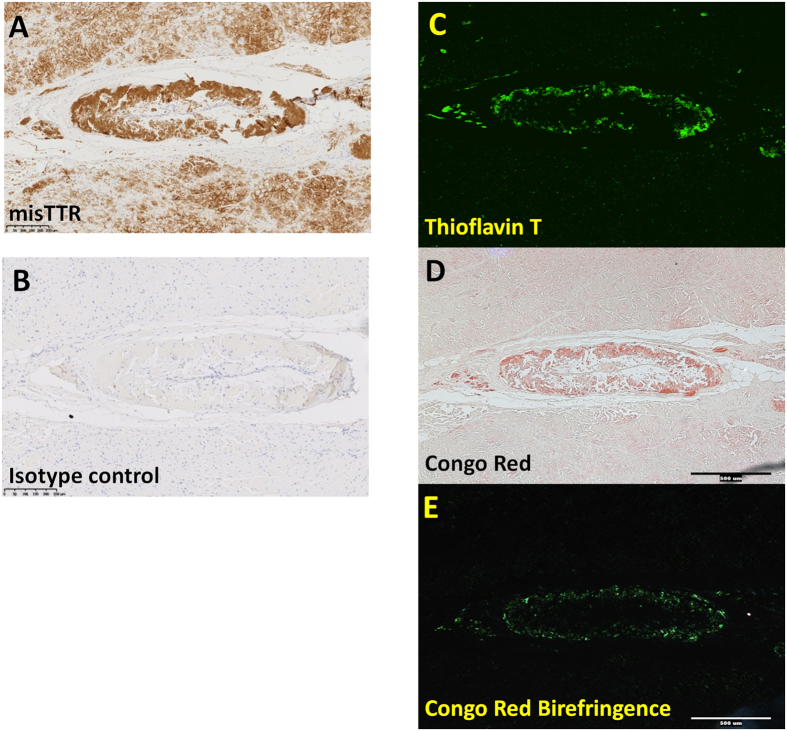
Misfolded TTR antibody labels TTR amyloids *in vivo*. Formalin fixed paraffin embedded (FFPE) cardiac tissue sections from a patient with I84S TTR mutation were labeled using misTTR (dark brown, **A**), a non-specific isotype control antibody (dark brown, **B**), or the amyloid specific dyes Thioflavin T or Congo Red (green **C**, red **D**, green **E**).

We then evaluated the reactivity of our anti-misfolded TTR antibodies and isotype controls by immunohistochemical analysis of cardiac tissue from validated cases of TTR amyloidosis. Cardiac amyloids were identified by staining using the dyes Thioflavin T and Congo red (Fig. 4C–E) and were confined primarily to the periphery of blood vessels found in these tissue sections. The misfolded TTR antibody selectively labels these Congo red-positive amyloid deposits with marginal labeling of Congo red-negative, non-amyloid TTR, whereas an isotype control antibody shows complete absence of immunoreactivity to the amyloid in the disease tissue (Fig. 4B).

### Misfolding-specific misTTR antibody reduces fibrillogenesis efficiency

**Figure 5:**
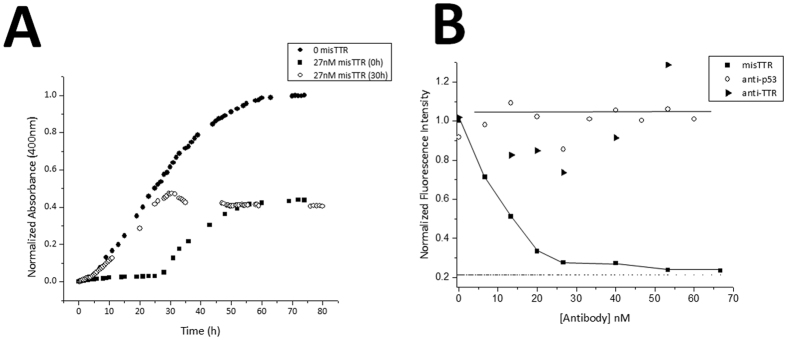
Effect of misTTR on TTR fibrillogenesis. (**A**) Time course of TTR fibril formation monitored by turbidity (3.6μM, 37 °C, pH 4.5). Substoichiometric amounts of misTTR were added at time 0 and after 30 hours to determine effect on TTR fibrillization process. (**B**) Dose dependent inhibition of TTR fibril formation by misTTR after 72 h. ThT emission peak was integrated from 475 nm to 495 nm and plotted versus antibody concentration (IC_50_ = 9.08 ± 0.32 nM).

In order to determine if misTTR antibody binding has an effect on TTR fibril formation, we evaluated the kinetics of the process using UV/VIS absorbance spectroscopy. A time course of TTR fibril formation revealed what appeared to be a nucleated polymerization mechanism, which is consistent with previously published reports^39^,^41^. A lag phase, corresponding to the length of time needed to form the amyloidogenic intermediate, was observed followed by a rapid growth phase, which began to plateau after 60 hours (Fig. 5A). In the presence of the misTTR antibody, however, a change in the kinetic profile of TTR fibrillogenesis occurred. There is an extended time period, lasting around 30 hours, where aggregates do not form, after which rapid aggregation occurs, but the aggregation level reaches only 40% of that seen in the absence of misTTR antibody (Fig. 5A). Thus it appears that the antibody changes the aggregation mechanism from being a well ordered multi-step process to a less efficient nucleation-mediated process.

Although the misTTR antibody was able to prolong the lag phase of TTR fibril formation, we looked to determine if it would have an effect when added to TTR after the fibrillogenesis process had started, namely during the polymerization or growth phase. The addition of substoichiometric amounts of misTTR antibody during the polymerization phase resulted in a slight decrease of the absorbance signal over a span of about 5 hours (Fig. 5A). While this slight decrease could be attributed to a dilution effect, this is highly unlikely due to the volume of antibody added. Since the observed signal decrease was gradual, it is likely that a slight disaggregation of prefibrillar species took place upon addition of the misTTR antibody.

In summary, the turbidity experiments highlight the complex inhibition of TTR fibrillogenesis, which includes a prolonged lag phase and apparent partial aggregation that plateaus at 40% of the level seen in the absence of antibody. To further explore this complexity a different technique was used to investigate the effect of the antibody on TTR fibrillogenesis.

### Substoichiometric amounts of the misTTR antibody suppresses TTR fibrillogenesis *in vitro*

We investigated the concentration dependence of misTTR inhibition using ThT fluorescence, a technique which measures the formation of amyloid fibrils. Physiological concentrations of TTR were left under fibril formation conditions in the presence of various concentrations of the misTTR antibody for 72 hours, and ThT was used to assess the extent of fibrillogenesis. Strikingly, a dose dependent inhibition of TTR fibrillogenesis was observed with IC_50_ = 9.08 ± 0.32 nM. Substoichiometric concentrations of the misTTR antibody were sufficient to suppress TTR fibrillogenesis *in vitro* (Fig. 5B). To confirm that the observed inhibitory effect is a consequence of the conformational specificity of the misTTR antibody, the experiment was repeated using the commercially available anti-TTR antibody. The pan-specific antibody, which displays no preference for native tetrameric TTR or misfolded conformations, had no effect on TTR fibrillogenesis, as assessed by ThT (Fig. 5B); it could neither stabilize the native fold of TTR nor prevent the dissociation and self-assembly of TTR into amyloid fibrils under the conditions used. As a third control, a polyclonal antibody directed against the tumor suppressor protein p53 also showed no effect. As such, we propose that the inhibition of TTR fibril formation is not a generic feature of polyclonal antibodies or even TTR antibodies, but likely stems from the design, specificity, and affinity of the misTTR antibody for monomeric, misfolded conformations of TTR present in the fibrillogenesis pathway.

To assess whether the misTTR polyclonal antibody was only effective at inhibiting wild-type TTR protein amyloidosis, the antibody was subject to a pH-induced aggregation experiment using the designed mutant, TTR Y78F, which exhibits misfolding and aggregation formation at a higher pH than wild-type TTR^42^. Results of this experiment showed the antibody to be just as effective at inhibiting mutant TTR aggregation (Supplementary Figure S4). These results indicate that the misTTR antibody was not only specific to the non-tetrameric, pathological misfolding intermediates of the normal TTR amyloidosis pathway, but also effective at inhibiting the aggregation of both wild-type and non-wild type TTR.

While the ThT fluorescence measurements indicate that the misTTR antibody completely inhibits TTR fibrillogenesis, the turbidity experiment revealed a complex inhibition involving a prolonged lag phase with partial aggregation reaching 40% of control. These differences are likely caused by the fact that ThT fluorescence measures amyloid fibril formation, while turbidity measurements gauge all forms of aggregation. The final aggregation products of these experiments may constitute a mixture of amyloid fibrils and nonspecific aggregates. Consequently, our results suggest that while this antibody inhibits TTR fibrillogenesis, it may not inhibit the formation of nonspecific aggregates.

### Proposed model of the inhibition of fibrillogenesis by the misfolding-specific misTTR antibody

The proposed mechanism by which misTTR antibody inhibits TTR fibrillogenesis is illustrated in Fig. 1A. Based on the data presented, we propose that the substoichiometric amounts of the developed antibody may act to suppress TTR fibrillogenesis in one of two ways. The first is as a disrupter of nuclei formation. During the course of fibrillogenesis, several TTR subunits associate to form a core or nucleus before cooperative growth can occur. However, since the association of subunits into a nucleus is an energetically unfavourable process, the lag phase generally persists for a period of time until a stable nucleus is formed. At a molar ratio of ~1:130 (antibody to TTR), misTTR antibody likely binds to small, oligomeric structures that are transiently populated during the lag phase and thereby delay the onset of polymerization. Binding to such structures may disrupt the assembly of stable nuclei needed for fibril growth. A second way by which the misTTR antibody may act to suppress TTR fibrillogenesis is by acting as a cap. At substoichiometric concentrations, misTTR antibody may prevent fibril formation by capping the ends of prefibrillar species and preventing growth.

**Figure 6:**
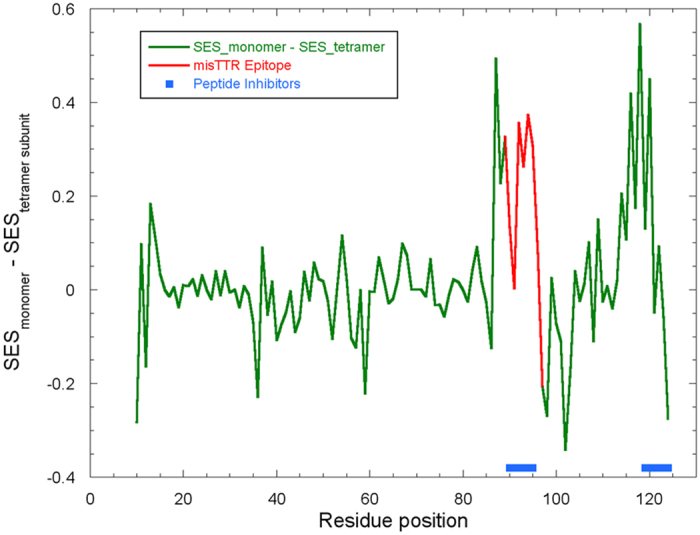
Correlation of solvent excluded surface area of tetrameric and monomeric TTR with peptide inhibitor segments and the misTTR epitope. The solvent excluded surface area (SES) of monomeric and the tetramer subunit form of TTR was calculated using Chimera molecular visualization program^47^ using the coordinate file 3KGU. The peptide inhibitors are those developed by Saelices *et al*.^43^. Note the correlation of the locations of the misTTR epitope and inhibitor peptides with changes in SES associated with monomerization of TTR.

In a recent publication, Eisenberg and coworkers^43^ report the development of two peptide-based inhibitors of TTR aggregation. These peptides were designed using the computational method ZipperDB^44^ to locate regions of the TTR sequence that had high fibril formation propensity. These peptide sequences were modified by substituting natural amino acids with N-methyl amino acids and by appending a tetra-arginine tail to either the N- or C-terminal. The peptides showed strong inhibition of TTR aggregation at concentrations above 20 μM. The first peptide corresponds to residues 91–96 of TTR. This segment is found right in the middle of our epitope (residues 89–97) (Fig. 6). This finding may be a coincidence or it may indicate that fibril formation initiation sites consist of buried segments of high fibril formation propensity. The fact that the peptide and the misTTR antibody both inhibited TTR fibril formation suggests that this region of TTR is exposed in misfolding intermediates and perhaps is also exposed on the growing ends of the fibril. The second peptide inhibitor is derived from residues 119–124 of TTR, which corresponds to the second region of TTR that is buried in the tetramer but exposed in the monomer (Fig. 6). We had previously examined an epitope peptide that includes this buried area (residues 105–120), but unfortunately that peptide did not elicit an immune response in rabbits and could not be investigated further. Combining our results with those of Eisenberg and coworkers^43^, we suggest that fibril initiation sites are particular segments of high fibril propensity that are buried in the native state, but become exposed in early, sparsely populated fibril intermediates, such as monomeric TTR. These sites appear to be ideal targets for both therapeutic antibodies and small molecule drugs.

### Concluding remarks

Using a structure-guided approach, we have designed the misTTR antibody which is able to distinguish between native and misfolded conformations of TTR. MisTTR is the first antibody to show inhibition of TTR fibrillogenesis, and more importantly, it achieves aggregation inhibition at low substoichiometric concentrations (IC_50_ = 9.08 ± 0.32 nM). Our findings suggest that this antibody has the potential to be used as a diagnostic tool in the identification of those patients with TTR amyloidoses. Furthermore, monoclonal versions of the misTTR antibody may have therapeutic potential for the treatment of FAP, hereditary ATTR, and wt-ATTR by acting as opsonization agents and facilitating clearance of both misfolded TTR and TTR aggregates using the mononuclear phagocytic system^45^. This work suggests there is value in investigating whether other oligomeric misfolding diseases involve the exposure of high-aggregation propensity segments in early stage monomeric misfolding intermediates.

## Methods

### Antibody generation

To generate the misTTR antibody, peptide synthesis was carried out using standard fluorenylmethoxycarbonyl (Fmoc)-based chemistry on a PS3 Automated Solid Phase Peptide Synthesizer (Protein Technologies Inc). A MAP with the following sequence, GGEHAEVVFTAGGKG, was synthesized on an [Fmoc-Lys(Fmoc)]4-Lys2-Lys-βAla-Wang resin (Advanced ChemTech, SM5102) using Fmoc-protected amino acids (Advanced ChemTech, Applied Biosystems, and Novabiochem). The peptide was subsequently cleaved from the resin with a mixture consisting of 90% trifluoroacetic acid, 8% anisole, and 2% triisopropylsilane (all from Sigma-Aldrich) and purified using ether extractions of protecting groups and scavengers; peptide composition was later verified using amino acid analysis. The MAP was then sent to Sigma-Genosys for rabbit antiserum production, which followed standard protocol (Sigma-Genosys) and was in accordance with the Animal Welfare Act (USA).

### Antibody purification

To purify the misTTR antibody from rabbit antiserum, a linear peptide with an identical sequence as the antigen used for antibody production was synthesized onto a non-cleavable TentaGel-SH resin (Advanced ChemTech). The resin was deprotected, acetylated, and packed into disposable columns (Evergreen Scientific). Columns were equilibrated with Phosphate-buffered saline containing 0.05% Tween-20 (PBS-T) before being used for rabbit antiserum purification.

For purification, rabbit antiserum (1 mL) was first pre-cleared by centrifugation (16,000 g), after which an equal volume of saturated ammonium sulfate was added. After one hour incubation at 4 °C, precipitate was recovered by centrifugation and washed several times with 50% saturated ammonium sulfate. The precipitate was then dissolved in PBS-T and added to the affinity purification TentaGel column. The column was subsequently incubated overnight at 4 °C, with end-over-end rotation, to allowing for binding to occur. The antibody-bound column was washed with PBS-T at room temperature until the wash eluent contained little or no protein (A_280_ = 0). Antibody fractions (1 mL) were eluted from column using 100 mM citric acid, pH 2.8, into tubes containing 250 μL of 1.5 M Tris and 150 mM NaCl, pH 8.0. The concentration of antibody in each fraction was determined using ultraviolet absorption spectroscopy and an IgG extinction coefficient at 280 nm of 1.35 (mg/mL)^−1^. The affinity purified antibody was stored at 4 °C and was generally stable for about 1 month.

### Protein expression and purification

A pET 21a (+) expression vector carrying TTR-(His)^6^ was transformed into Escherichia coli BL21-A1 competent cells (Invitrogen). Protein expression was induced with 1 mM isopropyl-β-D-thiogalactopyranoside when an absorbance (A_600_) of ~0.6 was reached. After 12–16 h incubation at 20 °C, cells were harvested by centrifugation at 5000 g. Pelleted cells were re-suspended in Buffer A (50 mM phosphate, 300 mM NaCl, 10 mM imidazole, and 20 mM β-mercaptoethanol, pH 8.0) and lysed by passage through an Emulsiflex-C5 (Avastin) for three cycles at 4 °C. After centrifugation at 27,000 g for 30 min at 4 °C, the supernatant was added to 5 mL of nickel-NTA (nitrilotriacetic acid) agarose slurry (Qiagen), gently mixed for 1 h, and loaded onto a column. The column was washed several times with Buffer B (50 mM phosphate, 300 mM NaCl, 20 mM imidazole, and 20 mM β-mercaptoethanol, pH 8.0) and the fusion protein was eluted using Buffer C (50 mM phosphate, 300 mM NaCl, 250 mM imidazole, and 20 mM β-mercaptoethanol, pH 8.0). The protein solution was dialyzed extensively against 10 mM phosphate, aliquotted, and frozen at −20 °C for later use.

The purity of the protein solution was confirmed by size exclusion chromatography. TTR eluted as a single peak at ~11 mL, representing tetrameric TTR, as shown in Supplementary Figure S2. Experiments were performed using recombinant TTR as well as wild-type TTR isolated from human plasma (Sigma-Aldrich). The biophysical properties of both plasma and recombinant TTR were found to be identical.

### TTR fibril formation assay

A stock solution of TTR (5 mg/mL) was diluted with 50 mM sodium acetate, pH 4.5, to a final concentration of 0.2 mg/mL. Samples were then incubated at 37 °C for 72 hours. The extent of fibril formation was probed by turbidity measurements at 400 nm on a Jasco V-500 UV-visible spectrometer equipped with a temperature control unit. Thioflavin T (ThT) was also used to assess the extent of fibril formation. In brief, a five-fold molar excess of ThT (Sigma-Aldrich) was added to each sample and left at room temperature for 30 minutes before measurements were taken. ThT fluorescence was monitored using a Photon Technology International C60 specrofluorimeter with the excitation and emission slit widths set to 4 nm. Spectra were obtained by scanning the fluorescence emission from 450 nm to 600 nm, with excitation at 442 nm.

### Enzyme-linked immunosorbent assay (ELISA)

To examine the ability of the developed antibody to discriminate between native and non-native conformations of the TTR, competition ELISA experiments were performed. For all assays, TTR unfolded in the presence of 6 M guanidine-HCl^37^ was used as bound antigen, while folded tetrameric TTR, monomeric TTR^39^, and TTR fibrils were used as competitors. In brief, a 96-well plate was coated with 100 ng of GdnHCl unfolded TTR per well and incubated overnight at room temperature (pH 4.5). Each well was washed three times with PBS-T and subsequently blocked with PBS + 1% bovine serum albumin (BSA) w/v (Sigma-Aldrich) for two hours. Various concentrations of competitor (folded TTR, monomeric TTR, or TTR fibrils) in the presence of 1 μg/mL of affinity purified misTTR or a commercially available anti-TTR antibody (Sigma-Aldrich) were added to wells and incubated for 1 h at room temperature. Wells were washed with PBS-T, and an HRP-conjugated anti-rabbit or anti-goat secondary antibody (1:5000) was added to wells and incubated at room temperature for 2 h. Wells were washed with PBS-T and 100 μL of the chromogenic substrate tetramethylbenzidine (Sigma Aldrich) was added. The reaction was stopped with 2M H_2_SO_4_, and an absorbance reading at 450 nm of the ELISA plate was taken immediately using a plate reader (SpectraMax M5, Molecular Devices).

All reported IC_50_ values were determined by fitting competition ELISA data to the following equation: 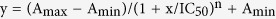.

### Western Blots

15% native-PAGE and SDS-PAGE gels were run using the protocol as outlined in Sechi and Chait’s publication in Analytical Chemistry (1998)^46^. However, 1/3 of the gel was removed and subject to coomassie staining as a visual reference. The remaining 2/3 of the gel were subject to immunoblots, with ½ of the remainder used for immunblotting with misTTR and the other ½ used for immunblotting with an anti-TTR rabbit antibody as a control (Sigma-Aldrich, #HPA002550) . Sample lanes consisted of 2 μg of lyophilized human TTR (Sigma-Aldrich) and was used Nitrocellulose membrane (Pall Life Sciences) was pre-soaked in PBS, pH 7.4 for 5 min and transferred into a PBS solution containing 20% methanol. The blot was blocked overnight at 4 °C with 5% skim milk, washed several times with PBS-T, and incubated at room temperature with affinity purified misTTR or anti-TTR antibody for 1 hour (1:1000). After several washes, the blot was incubated with IRDye^®^ 800CW Goat anti-Rabbit secondary antibody (Li-cor Biosciences, #925-32211) (1:5000) for 1 h, and imaged using LICOR^**®**^ Western Blot Analysis Software.

### Electron microscopy

The TTR sample in Fig. 3A was imaged using a Joel 1011 microscope operating at 80 kV. Samples were deposited on fresh continuous carbon films prepared from copper rhodium grids (Electron Microscopy Sciences). Grids were charged using a glow discharger for 15 seconds at 3 mA negative discharge before adding samples. Fibril solutions of 1 mg/mL were adsorbed to grids for 2 minutes before rinsing with 10 μL ddH20 for 10 seconds. Samples were then blotted using No. 2 Whatman Filter paper and stained with freshly filtered 2% uranyl acetate for 15 seconds.

Supplementary Figure S3’s TTR samples (0.2 mg/mL WT TTR in 140 mM NH_4_CH_3_COO, pH 4.5) were incubated with and without 70 nM misTTR antibody for 72 hours at 37 °C and 600RPM agitation. Samples were imaged using a Joel 1200 microscope operating at 80 kV at 1 hr, 24 hr, and 48 hr time points. Samples were deposited on carbon films prepared from copper rhodium grids (Electron Microscopy Sciences). Grids were charged using a glow discharger for 4–5 seconds at 3 mA prior to sample deposition. Samples were allowed 60 seconds for binding prior to wicking off with No. 2 Whatman Filter paper and then stained with freshly filtered 2% uranyl acetate for 10 seconds.

### Histology and Immunohistochemistry

Histochemical and immunohistochemical labeling was performed on 5 micron formalin fixed paraffin embedded sections of cardiac tissue from a patient with the TTR mutation I84S and confirmed ATTR amyloidosis. Congo red and Thioflavin T stains were used to demonstrate the presence of amyloid in the tissue. Congo red stain was performed using an alkaline Congo red solution (T3516-25G; Sigma-Aldrich, St. Louis, MO) following Pucthler’s modified protocol and Thioflavin T staining was performed with a filtered solution of 0.015% Thioflavin T (T3516-25G; Sigma-Aldrich, St. Louis, MO) in 50% ethanol.

Immunohistochemistry was conducted on a Leica Bond Rx (Leica Biosystems, Buffalo Grove, IL) autostainer using Leica’s proprietary Bond Polymer Refine Detection kit (DS980). The immunoperoxidase method was the principal detection system. The slides were incubated with 0. 5 μg/mL of the misTTR primary antibodies for 1 hour followed by incubation in anti-rabbit polymeric HRP-linker secondary antibody conjugates. The staining was visualized with a DAB chromogen, which produced a brown precipitate. The slides were counterstained with hematoxylin, dehydrated in an ascending series of alcohols, cleared in xylenes, and coverslipped with CytoSeal 60.

Negative controls consisted of performing the entire immunohistochemical procedure on adjacent sections of ATTR cardiac sections with a non-immune rabbit IgG isotype control and an omission of the primary antibody. Positive control slides included staining of sections of normal human choroid plexus and normal pancreas.

Slides were viewed with an Olympus BX61 microscope equipped with a 10x/0.40 and a 20x.0.75 UPlanSApo objective. Congo red-stained sections were evaluated under a cross-polarizer and analyzer while Thioflavin T was visualized in the fluorescence using appropriate FITC excitation and emission filters (Chroma Technology Corp., Bellows Falls, VT). Images were acquired with a Retiga Exi digital camera (QImaging; Surrey, BC) and imported with MetaMorph imaging system (Version 7.6.4.0; Molecular Devices; Sunnyvale, CA). All images were acquired and stored as Olympus Tiff files.

Whole-slides were digitally scanned with a Hamamatsu Nanozoomer 2.0HT slide scanner (Hamamatsu Corporation, Bridgewater, NJ) fitted with an Olympus 20x/0.75 UPlanSApo objective (Olympus).

## Additional Information

**How to cite this article**: Galant, N. J. *et al*. Substoichiometric inhibition of transthyretin misfolding by immune-targeting sparsely populated misfolding intermediates: a potential diagnostic and therapeutic for TTR amyloidoses. *Sci. Rep*. **6**, 25080; doi: 10.1038/srep25080 (2016).

## Supplementary Material

Supplementary Information
