# Corrigendum: Substoichiometric inhibition of transthyretin misfolding by immune-targeting sparsely populated misfolding intermediates: a potential diagnostic and therapeutic for TTR amyloidoses

**DOI:** 10.1038/srep27679

**Published:** 2016-06-10

**Authors:** Natalie J. Galant, Antoinette Bugyei-Twum, Rishi Rakhit, Patrick Walsh, Simon Sharpe, Pharhad Eli Arslan, Per Westermark, Jeffrey N. Higaki, Ronald Torres, José Tapia, Avijit Chakrabartty

Scientific Reports 6: Article number: 25080; 10.1038/srep25080 published online: 04282016; updated: 06102016

The Acknowledgements section in this Article is incomplete:

“This study was supported by a grant from the Canadian Institutes of Health Research to A.C and support from Prothena Inc. PW was supported by FAMY, FAMY Norrbotten and Amyl, and Erik, Karin and Gösta Selander’s Foundation. We thank Drs. D. Bateman and R. Wickner (National Institutes of Health, Bethesda, MD) for providing the pET 21a(+) expression vector used in this study”.

should read:

“This study was supported by the Ted Rogers Centre for Heart Research, a grant from the Canadian Institutes of Health Research to A.C and support from Prothena Inc. PW was supported by FAMY, FAMY Norrbotten and Amyl, and Erik, Karin and Gösta Selander’s Foundation. We thank Drs. D. Bateman and R. Wickner (National Institutes of Health, Bethesda, MD) for providing the pET 21a(+) expression vector used in this study”.

