# Supplementary material for: Substoichiometric inhibition of transthyretin misfolding by immune-targeting sparsely populated misfolding intermediates: a potential diagnostic and therapeutic for TTR amyloidoses
Source: Sci Rep. 2016 Apr 28;6:25080. doi: 10.1038/srep25080 (PMC4848561; doi:10.1038/srep25080)
Supplement: Supplementary Information [file srep25080-s1.pdf]

SUPPLEMENTARY MATERIALS

Substoichiometric inhibition of transthyretin misfolding by immune-targeting sparsely populated misfolding intermediates: a potential diagnostic and therapeutic for TTR amyloidoses

Natalie J. Galant[1*], Antoinette Bugyei-Twum[1*], Rishi Rakhit[2*], Patrick Walsh[3], Simon Sharpe[3], Pharhad Eli Arslan[1], Per Westermark[4], Jeffrey N. Higaki[5], Ronald Torres[5], José Tapia[5], and Avijit Chakrabartty[1,**]

1. Princess Margaret Cancer Centre, University Health Network, Departments of Medical Biophysics and Biochemistry, University of Toronto, TMDT 4-305, 101 College Street, Toronto, Ontario, Canada M5G 1L7
2. Department of Chemical and Systems Biology, Stanford University, CA 94305, USA
3. Molecular Structure and Function Program, the Hospital for Sick Children, Department of Biochemistry, University of Toronto, 1 King's College Circle, Toronto, Ontario, Canada M5S 1A8
4. Department of Immunology, Genetics and Pathology, Uppsala University, Uppsala, Sweden
5. Departments of Biochemistry and Histopathology, Prothena Biosciences Inc., South San Francisco, CA 94080

* Equal contributions
** Corresponding author: chakrab@uhnres.utoronto.ca; Tel: (416) 581-7553; Fax: (416) 581-7554

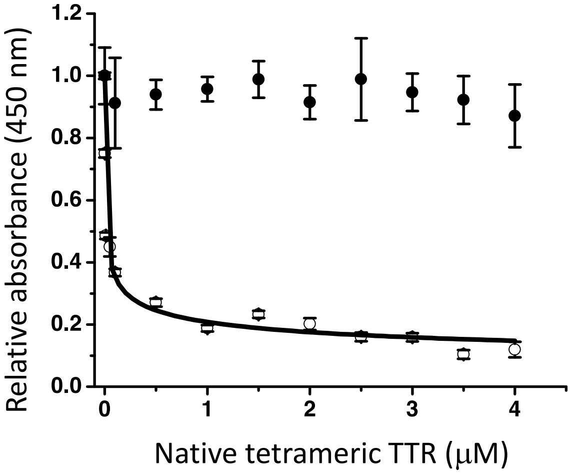

Figure S1. Binding specificity differences of commercial pan-specific TTR antibody (Sigma-Aldrich) (empty circles) and misTTR (filled circles). Competition ELISA using unfolded monomeric TTR as plate-bound antigen with native tetrameric TTR as competitor. While binding of commercial pan-specific TTR antibody (Sigma-Aldrich) to plate-bound unfolded TTR could be inhibited by native tetrameric TTR, binding of misTTR to plate-bound unfolded TTR could not be inhibited by native TTR.

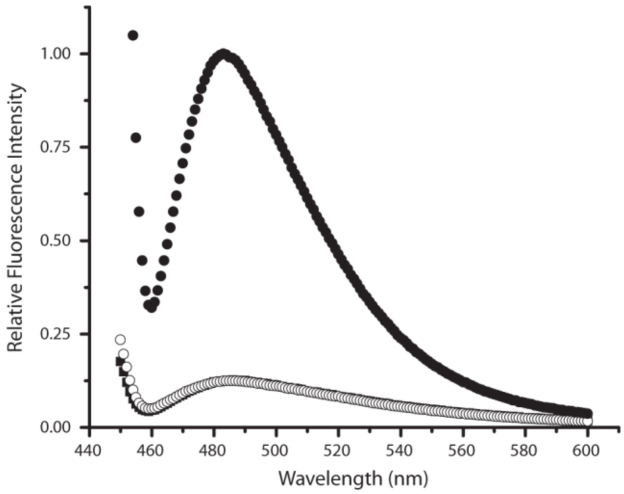

Figure S2. Fluorescence spectrum of TTR plus Thioflavin T (6-fold molar excess) at pH 4.5 (filled circles), and at pH 7.00 (empty circles). Fluorescence spectrum of TTR in 50 mM Tris, pH 7.00 (filled squares).

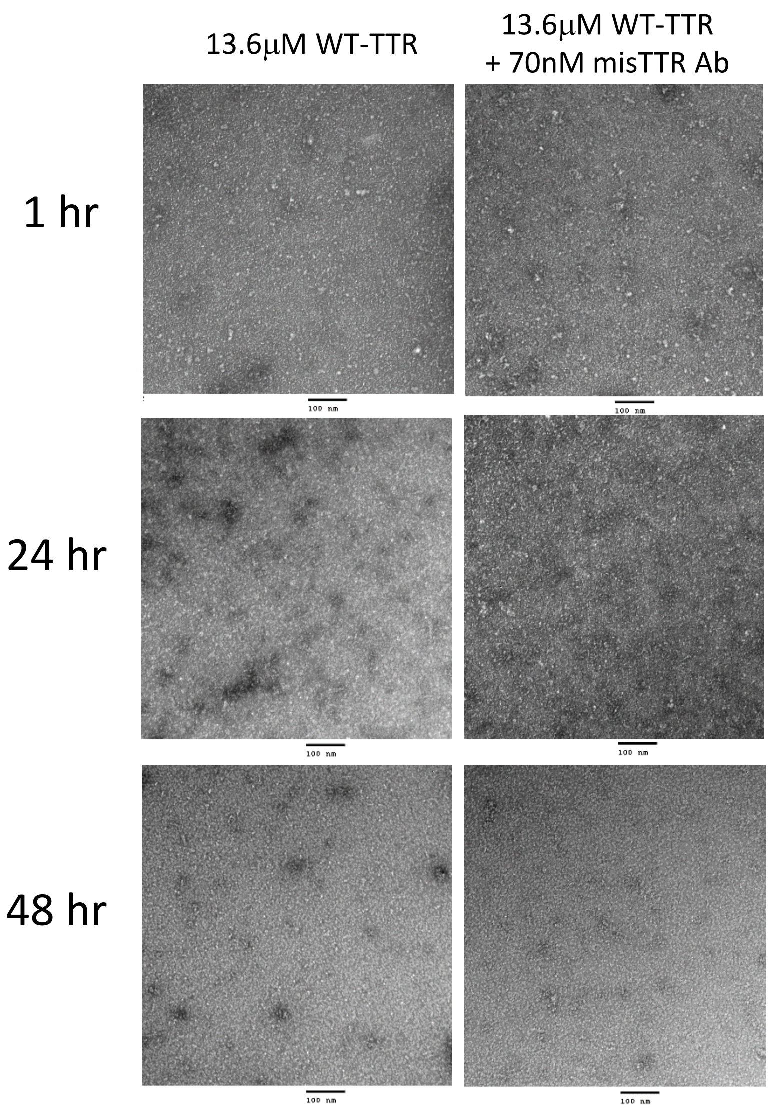

Figure S3. Transmission electron microscopy images of early stage *in vitro* TTR fibrillogenesis for 0.2mg/mL WT TTR (13.6μM) incubated with (RIGHT column) and without (LEFT column) 70nM misTTR antibody for 1hr, 24hrs, and 48 hours.

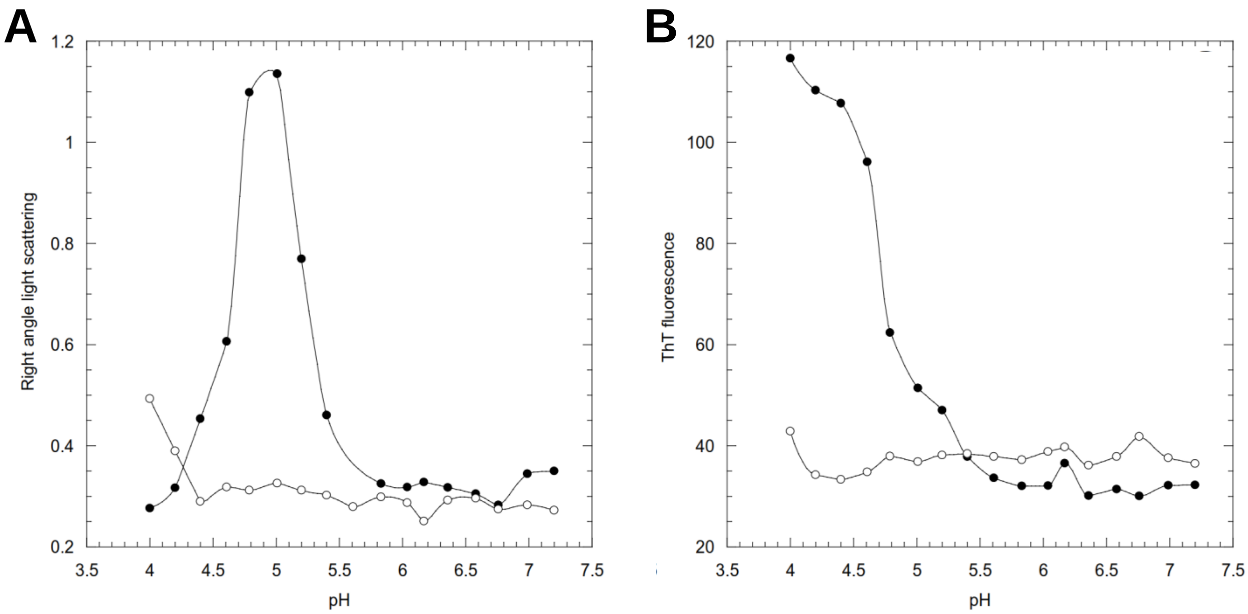

Figure S4. *In vitro* pH-induced aggregation profile of 2mg/mL Y78F TTR protein with (filled circles) and without (empty circles)1.4μM misTTR antibody as measured by right angle light scattering (350nm) (**a**) and Thioflavin T fluorescence (**b**). Both data sets indicate the misTTR polyclonal antibody to be effective at inhibiting the aggregation and amyloid formation of mutant TTR protein across a wide range of pH.
